# Manual therapy combined with core muscle training for chronic non-specific low back pain in sedentary individuals: a study protocol for a single-center randomized controlled trial

**DOI:** 10.3389/fmed.2026.1851073

**Published:** 2026-06-10

**Authors:** Dongming Su, Zihao Li, Yuan Xiong, Danni Xiong, Dan Yang, Jing Zhou

**Affiliations:** 1 School of Acupuncture and Orthopedics, Hubei University of Chinese Medicine, Wuhan, Hubei, China; 2 Department of Tuina, Rehabilitation Medicine Center, Hubei Provincial Hospital of Traditional Chinese Medicine, Wuhan, Hubei, China; 3 Affiliated Hospital of Hubei University of Chinese Medicine, Wuhan, Hubei, China; 4 Hubei Shizhen Laboratory, Wuhan, Hubei, China; 5 Hubei Key Laboratory of Theory and Application Research of Liver and Kidney in Traditional Chinese Medicine, Affiliated Hospital of Traditional Chinese Medicine, Wuhan, Hubei, China; 6 The First Clinical Medical School, Hubei University of Chinese Medicine, Wuhan, Hubei, China; 7 Hubei YJT Intelligent Technology Group Co., Ltd., Wuhan, China; 8 Hubei ZSK Medical Technology Co., Ltd., Ezhou, China

**Keywords:** chronic non-specific low back pain, core muscle training, manual therapy, randomized controlled trial, sedentary behavior

## Abstract

**Introduction:**

Chronic non-specific low back pain (CNLBP) represents a major global public health challenge, with sedentary behavior serving as one of its primary risk factors. Although clinical guidelines recommend core muscle training as a first-line intervention, a treatment dilemma often persists in clinical practice: the efficacy of training is constrained by pre-existing biomechanical, neurophysiological, and psychobehavioral barriers. This study proposes a novel two-stage synergistic model, “Passive Preparation and Active Re-education,” designed to overcome these bottlenecks and evaluate its effectiveness in a sedentary population.

**Methods and analysis:**

This is a single-center, assessor-blinded randomized controlled trial. A total of 88 sedentary individuals with CNLBP will be randomly allocated (1:1) to either a control group receiving core stability exercise alone or an intervention group receiving manual therapy followed by core stability exercise. The program consists of 12 sessions over 4 weeks. Outcome measures will be assessed at baseline, week 2, and week 4, with additional follow-ups at 1 and 3 months. The primary outcome is the change in pain intensity measured by the Visual Analog Scale (VAS) at week 4. Secondary outcomes include disability assessed by the Roland-Morris Disability Questionnaire (RMDQ), psychological factors via the Fear-Avoidance Beliefs Questionnaire (FABQ), and muscle biomechanical properties measured using MyotonPRO and isokinetic systems.

**Discussion:**

The central hypothesis is that the integration of manual therapy and core stability exercise produces a synergistic, non-linear effect rather than a simple additive one. Manual therapy acts as a “passive preparation” to create a time-sensitive “therapeutic window” by reducing pain and muscle hypertonicity. By immediately introducing core stability exercise within this window, the model optimizes target muscle recruitment and movement quality. This comprehensive approach aims to achieve a more efficient reversal of the pathological cycle in sedentary populations.

**Clinical trial registration:**

https://itmctr.ccebtcm.org.cn/mgt/, identifier ITMCTR2025002406.

## 1 Introduction

Chronic non-specific low back pain (CNLBP) is a syndrome presenting with pain and functional impairment localized between the lower margin of the ribs and the inferior gluteal folds, persisting for more than 12 weeks after specific pathologies (e.g., tumors, infections, or fractures) have been clinically excluded (1). In light of its long duration and high recurrence, CNLBP has emerged as a major global public health challenge. It is estimated that 15%–20% of the general population experience low back pain annually, and approximately 50%–80% will be affected at some point during their lifetime (2, 3). According to the Global Burden of Disease (GBD) study (4), this condition now stands as the leading cause of years lived with disability (YLDs) worldwide, accounting for 70.2 million YLDs in 2021, which represents 19.4% of total global YLDs and substantially compromises quality of life.

**FIGURE 1:**
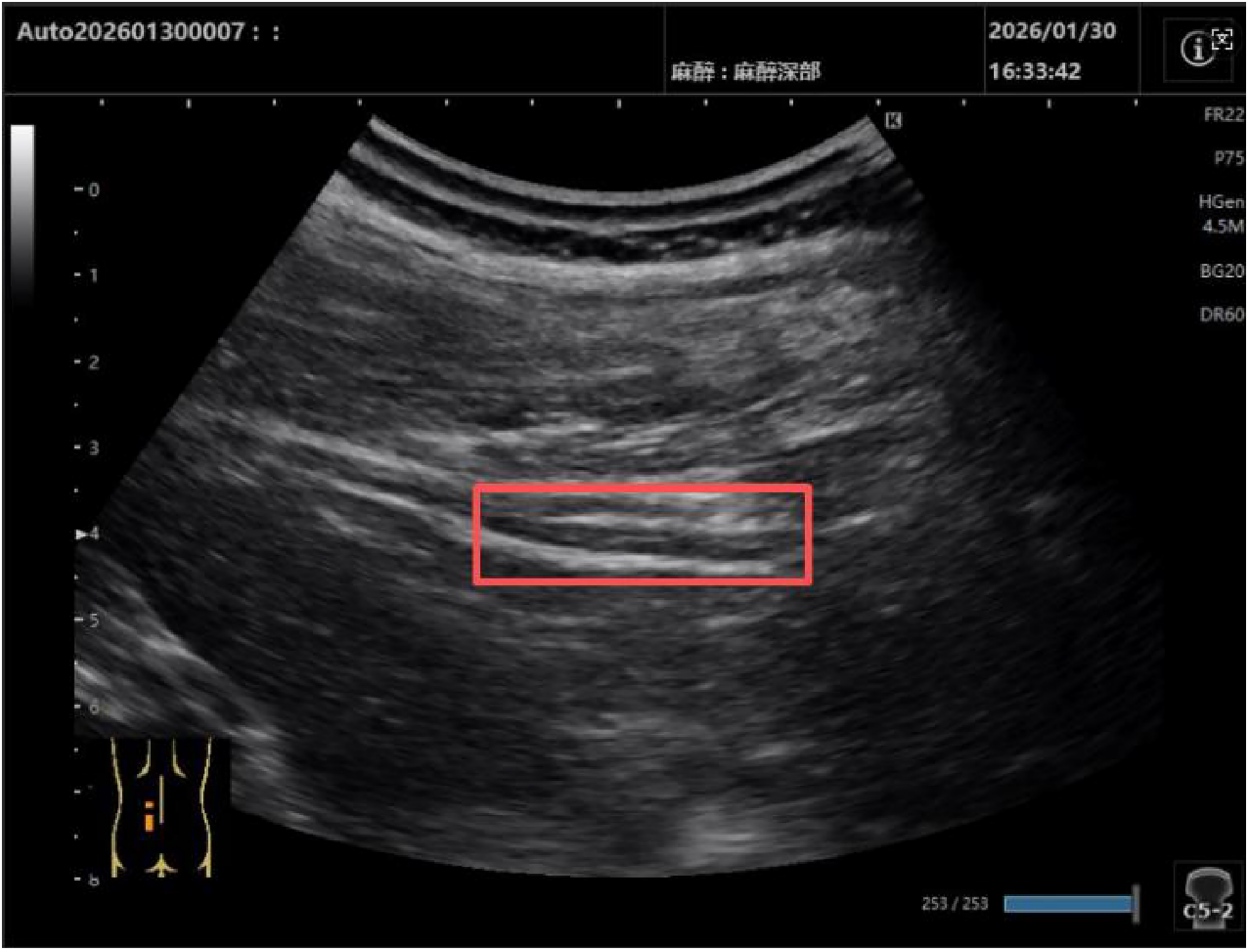
Muscle fiber changes on musculoskeletal ultrasound (SONIMAGE MXI PRO).

Sedentary behavior, defined as any waking behavior marked by a sitting, reclining, or lying posture with an energy expenditure of ≤1.5 metabolic equivalents of task (METs) (5), is considered one of the major risk factors for CNLBP. Specifically, prolonged sedentary behavior initiates and perpetuates a pathophysiological vicious cycle centered on dysfunction of the core musculature. The hallmark of this cycle is the overactivity of superficial muscles and the inhibition of deep muscles within the lumbar stabilization system. To maintain trunk stability during prolonged sitting, superficial muscles (e.g., erector spinae, quadratus lumborum) undergo sustained low-intensity static contraction. This persistent contraction results in pathological hypertonicity, subsequently triggering local tissue ischemia and the accumulation of metabolic waste products (6). If this state persists, it eventually induces the formation of myofascial trigger points (MTrPs), tissue adhesions, and even fibrotic changes (7), which can be visualized using musculoskeletal ultrasound (Figure 1).

Meanwhile, the deep stabilizing muscles, specifically the transversus abdominis and multifidus, which ensure fine segmental stability of the lumbar spine, experience functional inhibition and disuse atrophy in the absence of dynamic loading (8, 9). This pathological condition not only delays the feed-forward activation of these muscles but also markedly reduces overall neuromuscular control efficiency (10, 11). Additionally, the deep myofascia, notable for its deep-seated location and sparse microvascular supply, is prone to a self-perpetuating “ischemic-hypoxic-inflammatory” cycle driven by chronic mechanical overload and postural malalignment (12). This environment facilitates aberrant fibroblast activation and disorganized extracellular matrix (ECM) remodeling, culminating in the development of dense fibrotic adhesions (13, 14). Beyond acting as a mechanical obstruction that impairs microvascular perfusion and sequesters metabolic byproducts, including lactate and substance P (15), to drive peripheral sensitization, this pathological densification fundamentally compromises inter-layer sliding mechanics (11).

Based on the aforementioned pathophysiological mechanisms, major clinical guidelines consistently recommend exercise therapy centered on core muscle training as a first-line treatment for CNLBP (16, 17). The theoretical rationale underlying this approach is that active activation of deep stabilizing muscles can effectively correct dysfunctional core activation patterns and ultimately restore intrinsic spinal stability. Nevertheless, real-world clinical experience highlights a prominent treatment dilemma: while active core exercises improve functional outcomes, they often struggle to achieve clinically meaningful pain reduction on their own (18), as their efficacy is constrained by the very pathological conditions they seek to address. This dilemma primarily arises from three major barriers: (1) Biomechanical barriers: Elevated superficial muscle tension and myofascial adhesions disrupt joint kinematics, consequently leading to compensatory movements that impede precise deep muscle recruitment (19, 20). (2) Neurophysiological barriers: Persistent nociceptive input from pain signals induces reflexive motor neuron inhibition, thereby rendering effective neuromuscular activation difficult under pathological conditions (21), particularly in patients exhibiting altered pain processing or central sensitization (22). (3) Psychobehavioral barriers: Pain itself and negative expectations regarding pain provoked by movement predispose patients to fear-avoidance beliefs (23, 24). Such psychological distress is strongly associated with diminished muscle endurance and heightened functional impairment (25), which substantially reduce training adherence and self-efficacy (26). Collectively, the combined effects of these barriers contribute to delayed onset of therapeutic benefits and a higher incidence of early adverse responses during isolated core muscle training. Under such pathological interference, patients struggle to establish correct movement patterns, ultimately limiting clinical effectiveness. Consequently, identifying strategies to overcome these barriers and establish prerequisite conditions for active training has emerged as a critical bottleneck in improving rehabilitation outcomes for CNLBP.

To overcome these challenges, this study proposes a novel two-stage synergistic intervention model: “Passive Preparation and Active Re-education.” This model advocates that passive physical interventions, particularly manual therapy, should be administered as an enabling pre-treatment prior to active core exercises. Through precise mechanical stimulation, manual therapy effectively deconstructs the aforementioned barriers: (1) Relief of biomechanical constraints: Manual techniques reduce excessive superficial muscle tone, release myofascial adhesions, and improve tissue extensibility (27), thereby producing immediate increases in lumbar and hip range of motion (ROM). (2) Disruption of neurophysiological inhibition: By modulating the gate control mechanism and promoting the release of endogenous opioids, manual therapy achieves rapid analgesia (28, 29), which in turn markedly attenuates or abolishes pain-induced reflex inhibition of deep stabilizing muscles. (3) Reduction of psychobehavioral barriers: Immediate pain relief and a comfortable therapeutic experience enhance patients’ confidence and sense of safety (30), thereby improving acceptance of and adherence to subsequent core stability exercise.

Consequently, the central hypothesis of this study is that the combined application of manual therapy and core stability exercise does not constitute a simple additive effect, but rather a non-linear interaction characterized by synergistic enhancement. We propose that manual therapy, when implemented as “passive preparation,” creates an optimal “therapeutic window.” In this study, the “therapeutic window” is conceptualized as a short-term post-manual therapy state characterized by reduced pain, improved lumbar ROM, and decreased muscle stiffness, which may facilitate subsequent active core stability exercise. From both biomechanical and neurophysiological perspectives, this window substantially optimizes the prerequisites for subsequent active training. Conducting “active re-education” within this optimized physiological environment not only markedly improves movement quality and the efficiency of target muscle recruitment but also effectively increases training capacity. Through this synergistic mechanism, the intervention strategy may achieve a more rapid and more comprehensive reversal of the pathological vicious cycle underlying CNLBP.

## 2 Methods and analysis

### 2.1 Study design

**FIGURE 2:**
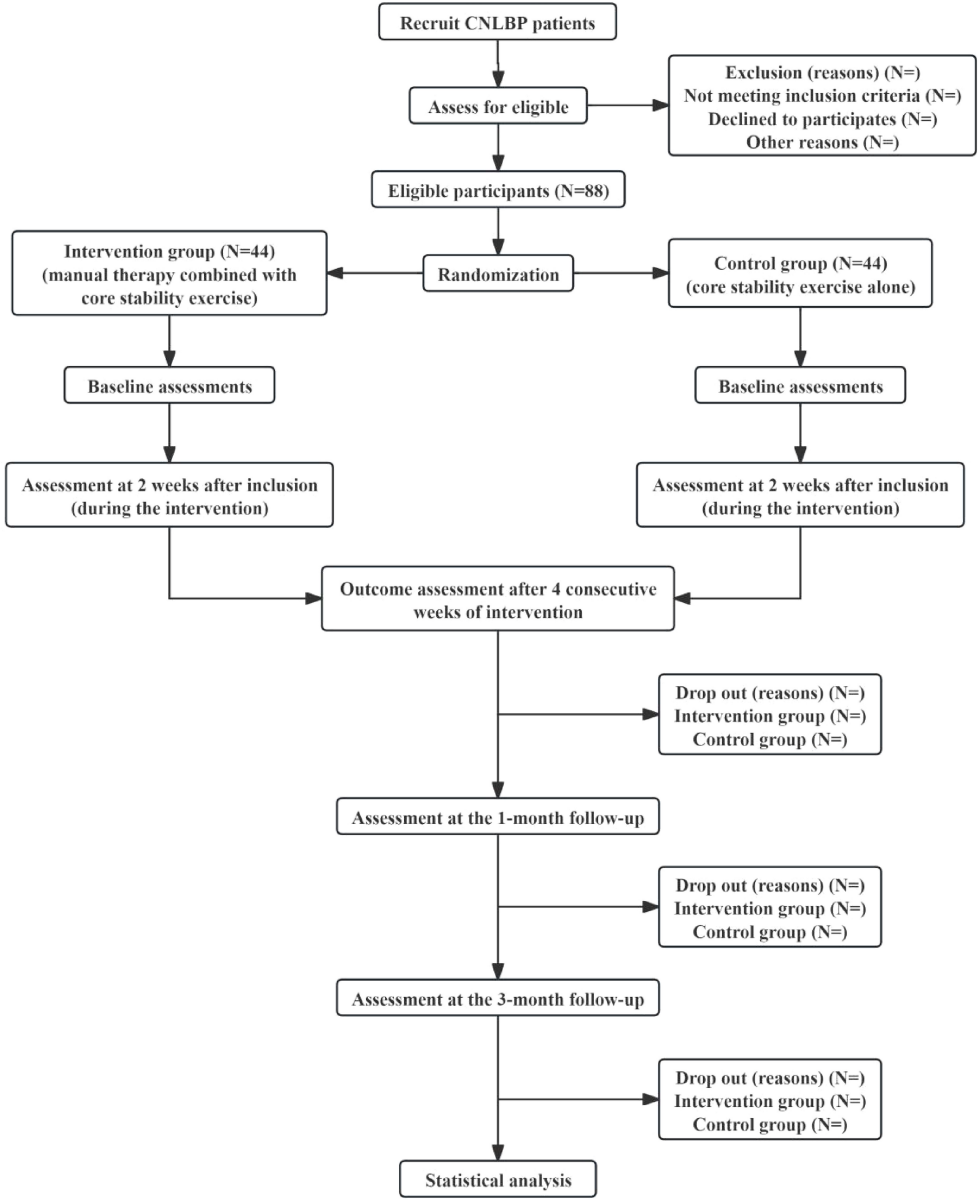
Trial flow diagram.

This single-center, open-label, assessor-blinded randomized controlled trial will be conducted from November 2025 to May 2026. The study was approved by the ethics committee of Hubei Provincial Hospital of Traditional Chinese Medicine, and all participants provided written informed consent. The trial adhered to the Declaration of Helsinki and is reported following the Consolidated Standards of Reporting Trials (CONSORT) guideline. A schematic flowchart is presented in Figure 2, and the study timeline is summarized in Table 1.

**TABLE 1:** Study design timeline illustrating enrollment, allocation, interventions, and outcome measurements at specified time points.

Time point	Enrollment	Allocation	Outcome measurement			
		Week 0	Week 2	Week 4	Month 2	Month 4
Enrollment						
Eligibility screen	●	–	–	–	–	–
Informed consent	●	–	–	–	–	–
Allocation	–	●	–	–	–	–
Interventions						
Core stability exercise	–	●	●	●	–	–
Manual therapy	–	●	●	●	–	–
Assessments						
Baseline characteristics	●	–	–	–	–	–
VAS	–	●	●	●	●	●
RMDQ	–	●	●	●	●	●
FABQ	–	●	●	●	–	–
MyotonPRO	–	●	●	●	–	–
ISOMED 2000	–	●	●	●	–	–
PPT	–	●	●	●	–	–
Adverse events	–	●	●	●	●	●

●, common in both groups; ●, experimental group only. VAS, Visual Analog Scale; RMDQ, Roland-Morris Disability Questionnaire; FABQ, Fear-Avoidance Beliefs Questionnaire; PPT, Pressure Pain Threshold.

### 2.2 Eligibility criteria

Participants will be recruited from the Rehabilitation Medicine Center/Tuina Department of Hubei Provincial Hospital of Traditional Chinese Medicine through outpatient screening and recruitment advertisements. A consecutive sampling strategy will be used: all potentially eligible sedentary individuals with chronic non-specific low back pain who attend the center during the recruitment period will be screened according to the eligibility criteria until the target sample size is reached. Sedentary behavior will be assessed using the sitting-time item of the International Physical Activity Questionnaire-Short Form (IPAQ-SF).

Participants will be eligible if they meet all of the following criteria:

(1) Aged 20–60 years;
(2) Diagnosed with chronic non-specific low back pain, defined as pain localized between the lower costal margin and the inferior gluteal folds lasting for more than 12 weeks, without identifiable specific pathology;
(3) Sedentary time of ≥6 h/day during a typical day;
(4) Baseline VAS pain score ≥ 3 on a 0–10 scale;
(5) No systematic physical therapy, manual therapy, acupuncture, or structured core muscle training within the previous 4 weeks;
(6) Able to understand the study procedures, provide written informed consent, and comply with the intervention and follow-up schedule.

Participants will be excluded if they have:

(1) Red-flag conditions, including suspected fracture, infection, tumor, unexplained weight loss, fever, or history of malignancy;
(2) Predominant radicular pain, progressive neurological deficit, or cauda equina syndrome;
(3) Spinal surgery or major spinal trauma within the past 3 months;
(4) Systemic inflammatory, rheumatic, neurological, or severe cardiopulmonary diseases;
(5) Severe osteoporosis, coagulation disorders, skin infection or open wounds in the treatment area, or other contraindications to manual therapy;
(6) Conditions preventing safe exercise participation;
(7) Pregnancy or lactation;
(8) Professional athlete status;
(9) Participation in another clinical trial.

### 2.3 Randomization, allocation concealment, and blinding

Following baseline assessments, participants will be randomly allocated in a 1:1 ratio. An independent researcher will generate the randomization sequence using a computerized random-number generator (SAS, version 9.1.3). To ensure allocation concealment, assignments will be placed in sequentially numbered, opaque, sealed envelopes. The treating therapist will open the next available envelope in front of each participant to reveal their group assignment.

All researchers, including outcome assessors and statisticians, will remain blinded to group allocation throughout the trial. The interventionists (manual therapist and core muscle exercise instructor) were necessarily unblinded but were strictly excluded from outcome assessment and data analysis to prevent bias. Participants will be instructed not to disclose their group allocation or treatment details to the outcome assessors during any assessment or follow-up visit.

### 2.4 Interventions

Participants will be allocated to either an intervention or a control group. The control group will engage exclusively in a 30-min core stability exercise program. In contrast, the intervention group received an additional 20-min manual therapy immediately prior to the core exercises. Both protocols will be administered three times per week for four consecutive weeks, totaling 12 treatment sessions.

In the intervention group, core stability exercise will begin immediately after the 20-min manual therapy session, following a brief safety check and participant feedback. The “therapeutic window” is not used as a mandatory criterion for initiating exercise; therefore, participants will proceed with the planned exercise unless safety concerns or intolerable pain are reported.

The manual therapy served as a preparatory protocol, designed to reduce myofascial tension and enhance neuromuscular readiness for the subsequent core stability exercises. The intervention will be delivered by a senior licensed Traditional Chinese Medicine (TCM) therapist with over a decade of clinical experience. The intensity will be individualized by integrating the therapist’s clinical judgment with findings from the physical examination and the participant’s self-reported response. Specifically, manual pressure will be titrated based on three criteria: (a) maintaining a pain tolerance score between 4 and 7 on a 0–10 scale; (b) reaching the perceived tissue resistance barrier; and (c) using a real-time verbal feedback mechanism for dynamic adjustments during each session. The protocol will systematically follow a three-phase standard operating procedure (SOP) (Supplementary Videos 1, 2 and Supplementary Figure 1):

Phase 1: Superficial relaxation (5 min): employing “palm-rubbing” and “pressing” techniques (using the palm or thumb to apply rhythmic circular pressure) on the lumbodorsal musculature. Phase 2: Deep tissue release (10 min): utilizing thumb acupressure and “plucking” techniques to address MTrPs and nodules within the erector spinae and quadratus lumborum. “Plucking manipulation” is defined as repeated transverse thumb pressure applied perpendicular to the muscle fibers to mechanically stimulate MTrPs, nodules, and areas of increased tissue resistance. Phase 3: Transitional mobilization (5 min): concluding with passive stretching of the lumbar spine.

The 30-min core stability exercise program, supervised by a professional rehabilitation therapist, was structured progressively across three stages (Supplementary Figure 2). Stage 1 focused on activating deep stabilizers (e.g., transverse abdominis contractions, pelvic tilts, bird-dog exercise). Stage 2 aimed to strengthen global movers (e.g., plank, bridge). Finally, Stage 3 involved the integration of functional movements. Throughout this process, the criteria for progression will include: (1) pain-free completion of current stage exercises; (2) correct muscle activation verified by therapist; (3) patient-reported confidence ≥ 7/10.

In this protocol, the “therapeutic window” is used as a conceptual and mechanistic framework rather than as a mandatory criterion for treatment progression. In the intervention group, core stability exercise will begin immediately after the 20-min manual therapy session, following a brief safety check and participant feedback. If a participant does not achieve a ≥ 30% reduction in VAS after manual therapy, the planned core stability exercise will still be performed, provided that no safety concerns or intolerable pain are reported. Exercise intensity will be adjusted according to the participant’s pain tolerance and the therapist’s clinical judgment.

### 2.5 Outcome measurement

All outcome measures will be assessed at baseline, the end of week 2, and the end of week 4. Additionally, pain intensity and disability were re-assessed at the 1-month and 3-month follow-ups.

#### 2.5.1 Primary outcome

The primary outcome is the change from baseline in pain intensity at week 4. Pain intensity will be assessed using the 10-cm Visual Analog Scale (VAS), with 0 indicating “no pain” and 10 indicating “the worst imaginable pain.” The VAS is widely used in low back pain research and has demonstrated acceptable reliability, validity, and responsiveness for musculoskeletal pain assessment (31). A reduction of 2 points is commonly interpreted as clinically meaningful improvement. A 2-point difference on the VAS was defined as the minimal clinically important difference (MCID) (32).

#### 2.5.2 Secondary outcome

##### 2.5.2.1 Disability

The Roland-Morris Disability Questionnaire (RMDQ) will be used to evaluate low back pain-related disability. It contains 24 items, with higher scores indicating greater disability. The RMDQ has been widely validated in patients with low back pain and is sensitive to functional changes over time (33). The MCID values for RMDQ are 5 points (34).

##### 2.5.2.2 Psychological factors

Fear-avoidance beliefs will be evaluated using the Fear-Avoidance Beliefs Questionnaire (FABQ). The FABQ comprises two independent subscales and includes a total of 16 self-report items. The FABQ-Phys, which contains five items and has a total score range of 0–24, is intended to assess fear-avoidance beliefs related to general physical activities. The FABQ-Work, comprising 11 items with a total score range of 0–42, is employed to evaluate fear-avoidance beliefs associated with occupational activities. A higher score indicates a higher level of fear-avoidance beliefs (35). The MCID values for FABQ-Work and FABQ-Phys are seven and four points, respectively (36).

##### 2.5.2.3 Biomechanical properties

Muscle viscoelasticity will be assessed using the MyotonPRO device (37). The measurement points will be selected bilaterally at 1.5 cm lateral to the L3 spinous process. Measurements will be performed by a trained assessor under standardized room temperature and participant positioning. Three repeated measurements will be taken at each measurement point, and the mean value will be used for analysis. The specific parameters will include frequency (Hz) and stiffness (N/m), reflecting baseline muscle tone and muscle stiffness, respectively.

The strength and endurance of the lumbar flexor and extensor muscles will be assessed using the ISOMED 2000 system according to a standardized protocol. Five maximal-effort concentric contractions of lumbar flexion and extension will be performed at angular velocities of 60 and 120°/s, respectively. Participants will complete a familiarization test before the formal assessment. The test data will include peak torque, total work, and endurance index. Adequate rest intervals will be provided between trials to reduce fatigue effects.

##### 2.5.2.4 Sensory-physiological properties

Pressure Pain Threshold (PPT, kg/cm^2^) will be measured using a digital pressure algometer at the most tender point of the erector spinae. Pressure will be increased at a constant rate until the participant first reports pain. Three measurements will be performed at 30 s intervals, and the mean value will be used for analysis.

### 2.6 Safety evaluation and adverse events (AEs)

Continuous safety monitoring of participants will be required during each intervention session throughout the trial. All AEs occurring during the intervention or follow-up period will be systematically recorded in the case report forms (CRFs) and carefully evaluated for their potential association with manual therapy or core muscle training. Potential risks associated with manual therapy or core muscle training include aggravation of localized pain, muscle or joint injuries during exercise, dizziness, extreme fatigue, and other post-intervention discomforts. Should an AE occur — regardless of its relationship to the study — qualified medical professionals will provide immediate symptomatic treatment. Investigators will complete detailed documentation in the CRFs regarding the onset time, clinical manifestations, severity grading, therapeutic measures implemented, and clinical outcomes of the AE. Safety monitoring will continue until resolution or stabilization of the participant’s condition. All AEs will be reported to the principal investigator (PI) and the institutional review board (IRB) for review, followed by an assessment of the participant’s eligibility for continued participation. Compensation procedures will be activated for study-related serious adverse events (SAEs) in accordance with regulatory requirements and institutional policies.

### 2.7 Data management and quality control

Data collected by qualified physicians will be stored in a dedicated electronic database, with participants’ identifying information anonymized using unique numerical codes. The electronic case report form (eCRF), designed in accordance with the Ethics Committee-approved template, will serve as the primary instrument for clinical data collection. Data processing will adhere to the principles of Good Clinical Practice (GCP) and comply with applicable Chinese regulatory requirements. Access to the electronic database system will be restricted to authorized researchers through role-based permissions and password protection to ensure the confidentiality and traceability of all entries. To enhance participant compliance, researchers will provide timely reminders via telephone for scheduled visits and assessments. For subjects who withdraw from the clinical trial or follow-up, detailed reasons and outcome records will be systematically documented.

### 2.8 Sample size calculation

The sample size was calculated using G*Power software (version 3.1.9.7) based on the change in the primary outcome measure, the VAS scores. Because few previous trials have directly compared manual therapy combined with core stability exercise versus core stability exercise alone in sedentary individuals with CNLBP, the sample size was estimated using the best available evidence from a randomized trial evaluating exercise-based interventions for chronic non-specific low back pain (38). The expected between-group difference in VAS was set at 1.5 points, with a pooled standard deviation of 2.3, corresponding to Cohen’s *d* = 0.65. This effect size was considered clinically plausible and consistent with a moderate treatment effect in musculoskeletal rehabilitation.

In this study, the type I error probability (α) for a two-tailed test was set at 0.05, and the statistical power (1−β) at 80%. Based on these parameters, a total sample size of 74 participants is required. Accounting for a potential 15% dropout rate, the adjusted sample size per group is 44, resulting in a total planned recruitment of 88 participants.

### 2.9 Statistical analysis

Baseline and efficacy analyses will be conducted following the intention-to-treat (ITT) principle. The distribution of continuous variables will be assessed using visual inspection of histograms and Q-Q plots, together with the Shapiro-Wilk test. Homogeneity of variance will be examined using Levene’s test. Normally distributed continuous variables will be presented as mean ± standard deviation and compared using independent-samples *t*-tests, whereas non-normally distributed variables will be presented as median and interquartile range and compared using the Mann-Whitney U test. Categorical variables will be summarized as frequencies and percentages and compared using the chi-square test or Fisher’s exact test when expected cell counts are small.

Linear mixed-effects models (LMMs) will be used to assess efficacy. The model will include group, time, and their interaction as fixed effects, with participants as a random effect, adjusting for baseline values. For linear mixed-effects models, assumptions will be checked using residual and Q-Q plots. If substantial violations are identified, appropriate transformations or robust sensitivity analyses will be considered. The group-by-time interaction term will be used to test for between-group differences in the change from baseline for both the primary (VAS) and all secondary outcomes at weeks 2 and 4. This term was also used to assess group differences in VAS and RMDQ scores at both the 1- and 3-month follow-ups. Additionally, a supportive analysis compared clinical response rates at week 4 using a chi-square test or logistic regression.

A sensitivity analysis will be repeated the primary analysis on the per-protocol (PPS) set. The LMMs inherently handles missing data; multiple imputation was used for other analyses where necessary. Exploratory correlations between outcome changes will be assessed using Pearson correlation for normally distributed variables and Spearman correlation for non-normally distributed or ordinal variables.

## 3 Discussion

Due to the potential adverse effects and addiction risks associated with pharmacological interventions, there is a growing demand for safer and more effective treatment strategies for CNLBP (39). Consequently, non-pharmacological approaches have gained significant attention. Among these, manual therapy serves as a passive intervention to improve joint mobility and modulate pain, while core muscle training acts as an active strategy to enhance spinal stability and muscular control. While existing trials demonstrate preliminary efficacy (40, 41), high-quality RCTs with standardized protocols remain limited. Therefore, we have meticulously designed this single-center randomized controlled trial to evaluate the effectiveness of manual therapy and core muscle training in patients with CNLBP.

The “three-phase” manual therapy protocol designed in this study derives its core value from the precise removal of tissue obstructions through graded mechanical stimulation. From a biomechanical perspective, the “plucking” and “pressing” techniques applied to the deep myofascia are intended not only to release adhesions but also to improve matrix viscoelasticity via shear forces (42). This process aims to restore impaired inter-layer sliding function, thereby providing the physical prerequisites for the precise spinal control required during subsequent core stability exercise. On a neurophysiological level, manual therapy achieves rapid analgesia by activating the “gate control” mechanism. This effectively attenuates the reflexive inhibition of deep stabilizing muscles induced by pain. Research indicates that this neuromuscular disinhibition effect is a biological prerequisite for the precise recruitment of deep muscle groups (43).

We hypothesize that combining manual therapy with core stability exercise yields synergistic, non-linear effects exceeding simple additive benefits. Manual therapy serves as “passive preparation,” creating a time-limited “therapeutic window” characterized by reduced pain and muscle hypertonicity, within which immediate core exercise optimizes motor learning and neuromuscular recruitment. By leveraging the biomechanical benefits provided by this pre-treatment and immediately introducing active re-education, the model prevents the “rebound” effect often seen after passive intervention alone (44). This approach significantly lowers the entry barrier for core stability exercise, allowing patients to capture correct movement sensations in a pain-free or low-pain state. This coupling mechanism is designed to achieve more efficient neuromuscular remodeling than monotherapies, thereby more thoroughly reversing the pathological cycle of core imbalance.

Psychobehavioral barriers represent a critical bottleneck affecting long-term adherence to CNLBP rehabilitation (45). This protocol suggests that the immediate analgesia provided during the passive phase is not only a physiological benefit but also a cognitive catalyst for rebuilding patient self-efficacy. For patients with “fear-avoidance” beliefs, early experiences of comfortable and effective treatment can rapidly disrupt negative associations between movement and pain (46). This positive physical feedback significantly enhances patient acceptance of subsequent core stability exercise. Consequently, by optimizing both psychological and physiological factors, this model transforms patients from “passive recipients” to “active participants” in their recovery, serving as the core link for ensuring the precise execution of complex rehabilitation protocols.

In conclusion, the synergistic model proposed in this study offers a novel approach to addressing clinical rehabilitation bottlenecks in CNLBP. Specifically tailored to the pathological characteristics of sedentary populations, the protocol ensures intervention reproducibility through a systematic SOP.

Nevertheless, this study has certain limitations. First, it is a single-center trial with a relatively modest sample size, which may limit the generalizability of the findings. As the sample size was calculated based on the primary pain outcome, analyses of secondary biomechanical outcomes should be interpreted cautiously. Second, due to the nature of the intervention, therapist blinding was not feasible. Another limitation is the unequal therapist-contact time between groups, as the intervention group will receive an additional 20-min manual therapy session. Thus, potential attention bias cannot be completely excluded. To minimize non-specific differences, both groups will receive standardized instructions and the same follow-up schedule. Future studies could involve multi-center, large-sample trials and utilize functional imaging techniques to investigate the cortical remodeling mechanisms involved in this “re-education” process. Additionally, exploring the application of this model in other musculoskeletal pain conditions would have significant clinical value.
