# Supplementary material for: Manual therapy combined with core muscle training for chronic non-specific low back pain in sedentary individuals: a study protocol for a single-center randomized controlled trial
Source: Front Med (Lausanne). 2026 Jun 10;13:1851073. doi: 10.3389/fmed.2026.1851073 (PMC13290618; doi:10.3389/fmed.2026.1851073)
Supplement: Supplementary file 3 [file Image_1.pdf]

Supplementary material

Manual therapy combined with core muscle training for chronic non-specific low back pain in sedentary individuals: a study protocol for a single-center randomized controlled trial

Dongming Su, ZiHao Li, Yuan Xiong, Danni Xiong, Dan Yang*, Jing Zhou*

Supplementary Video 1: Palm-rubbing and Pressing Techniques

This video demonstrates the palm-rubbing and pressing techniques applied to the lumbodorsal musculature for superficial relaxation. The therapist uses the palm or thumb to apply rhythmic circular pressure, aiming to reduce muscle tension.

Supplementary Video 2: Plucking Technique

This video illustrates the plucking technique targeting myofascial trigger points and nodules in the erector spinae. The therapist applies transverse pressure across the muscle fibers to release deep adhesions.

Supplementary Figure 1: Passive Spinal Stretching

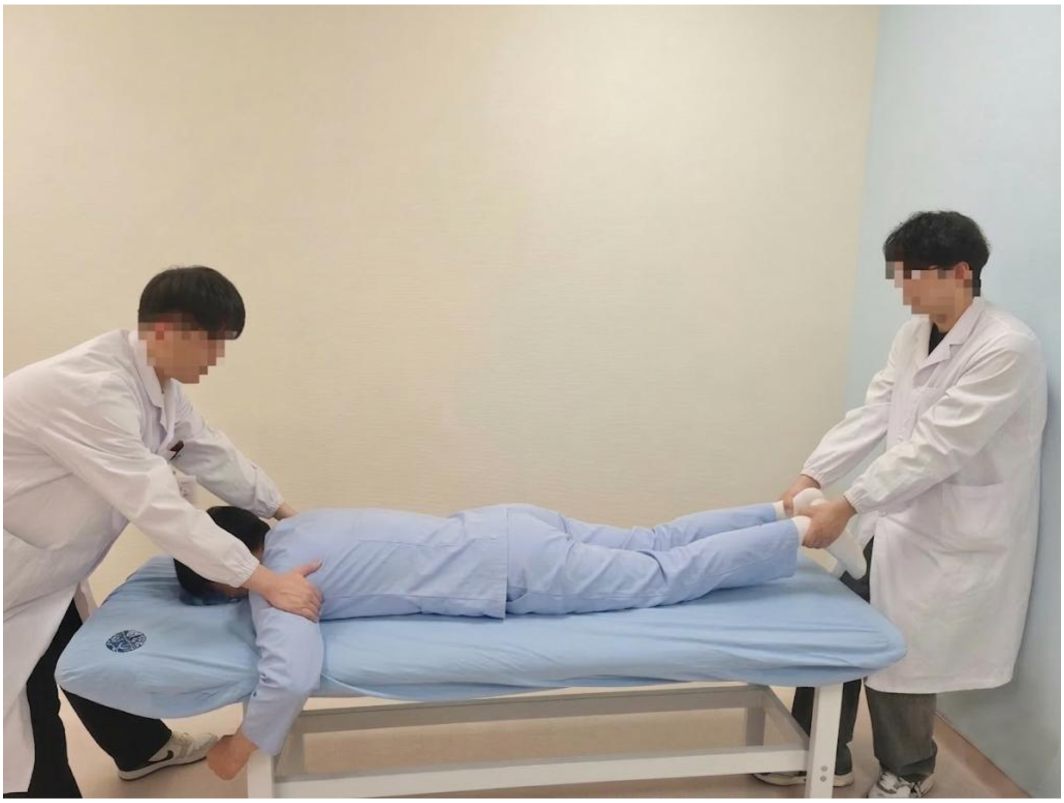

A demonstration of the passive traction technique. This longitudinal stretching aims to decompress the intervertebral spaces and improve spinal mobility during the transitional mobilization phase.

Supplementary Figure 2: Stages of the Core Stability Exercise Program

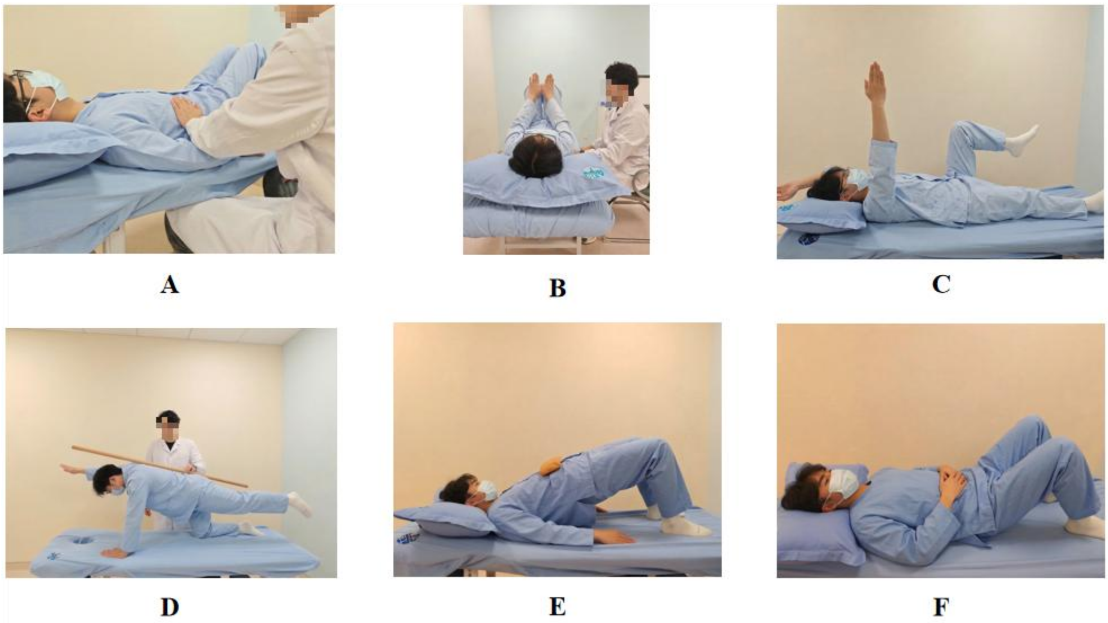

(A) Abdominal Drawing-in Maneuver: Activating the transversus abdominis in a supine position.

(B) Pelvic Tilt: Demonstrating the control of pelvic neutral alignment.

(C) Dead Bug Exercise: A Stage 1 exercise for enhancing lumbopelvic stability during limb movement.

(D) Bird-dog Exercise: Using a wooden stick to ensure a neutral spine and trunk alignment during the movement.

(E) Bridge Exercise: A Stage 2 exercise focusing on strengthening the posterior chain muscles.

(F) Abdominal Breathing: Active regulation of Intra-abdominal pressure and diaphragmatic displacement to optimize respiratory efficiency.
